# Identification of inverted U-shaped curve association between serum potassium and prodromal Parkinson’s disease

**DOI:** 10.1016/j.prdoa.2025.100323

**Published:** 2025-04-09

**Authors:** Wen Zhou, Qingqing Xia, Duan Liu, Jun-ying Li, Liang Gong

**Affiliations:** Chengdu Second People’s Hospital, No. 10, Qingyun South Street, Jinjiang District, Chengdu 610017 Sichuan, China

**Keywords:** Prodromal Parkinson’s disease, Serum potassium, Inverted U-shaped, Retrospective cross-sectional study

## Abstract

**Background:**

The association between serum potassium and prodromal Parkinson’s disease (PPD) remains unclear currently.

**Objective:**

The ultimate goal is to gain a deeper understanding of the implications of this association between serum potassium and PPD.

**Methods:**

We conducted a retrospective cross-sectional study involving 1035 participants in PPMI cohort. Age, sex, education years, race, body mass index (BMI), calcium, alanine aminotransferase, aspartate aminotransferase, lymphocytes, neutrophils, serum uric acid, serum sodium, serum potassium, creatinine, serum glucose were obtained from all participants. Logistic regression, and smooth curve fitting were utilized to substantiate the research objectives.

**Results:**

The overall PPD was 83.4 % (863/1035). Multivariate odds ratio regression adjusted for risk factors demonstrates a 1-unit increment in the serum potassium raises the risk of PPD by 1.82 times, respectively. Smooth splines analysis suggested an inverted U-shaped association between serum potassium and PPD (P nonlinearity < 0.05), with the zenith of risk at 4.479 mmol/L. Further subgroup analysis and sensitivity analyses supported the primary findings and indicated the conclusions are robust.

**Conclusions:**

This study highlights the association between serum potassium levels and the risk of incident PPD, independent of confounders. The association between serum potassium and PPD is inverted U-shaped, the threshold value is 4.479 mmol/l.

## Highlights

• This paper shows for the first time that serum potassium and prodromal Parkinson’s disease (PPD) are associated.
• After controlling for confounding factors, we still found an association between serum potassium and PPD.
• Smoothed plots show an inverted U-shaped association between serum potassium and PPD, with a critical threshold at 4.479 mmol/l.
• Subgroup analysis and sensitivity analysis were performed in this study, and the results remained robust.

## 1 Introduction

Parkinson's disease (PD) is marked by a progressive array of motor impairments that typically emerge in later life. The pathogenesis of PD is believed to begin well before the clinical manifestation of the disease as currently defined. Prodromal Parkinson's disease (PPD) refers to a preclinical phase where individuals exhibit an increased risk of developing PD due to clinical manifestations, genetic predispositions, or biomarker presence, but have not yet received a formal diagnosis [1].

Potassium is a critical electrolyte essential for nerve and muscle function. A recent cross-sectional study demonstrated elevated serum potassium levels in patients with PD compared to healthy controls [2]. This elevation may be closely related to the formation of neurotoxic α-synuclein oligomers, which are believed to play a pivotal role in the neuropathogenesis of PD [3]. Additionally, fluctuations in potassium ion concentration can influence copper ion uptake via a Cu^+^/2K^+^ antiport mechanism observed in yeast models. Imbalances in copper homeostasis can exacerbate α-synuclein aggregation [4].

These findings underscore the importance of investigating the relationship between serum potassium and PPD. Establishing this link may facilitate the development of simple, cost-effective population-based screening methods for PPD, enabling targeted neuroprotective interventions and improve outcomes for patients at risk of developing PD. Our current investigation aims to examine the potential association between serum potassium levels and PPD.

## 2 Methods

### 2.1 Participant selection and study grouping

The Parkinson's Progression Markers Initiative (PPMI) study comprehensively assessed clinical, imaging, and biochemical parameters in individuals with PPD and healthy controls, with data extracted from the PPMI database on January 4, 2024. The analysis was confined to baseline data collected at the time of participant enrollment (Supplementary Fig. 1). Eligibility for the PPD cohort was determined using a tiered risk assessment, with the following criteria:

Age: ≥60 years; however, individuals ≥30 years with SNCA or other rare genetic mutations (e.g., PRKN, PINK1) are included.
Clinical Diagnosis: No diagnosis of PD, parkinsonian syndromes, or dementia. Inclusion is based on PD risk factors or features such as REM sleep behavior disorder (RBD), genetic risk variants, family history of PD, or criteria from PPMI Online questionnaires.
Hyposmia Assessment: University of Pennsylvania Smell Identification Test (UPSIT) scores indicating hyposmia are required, except for those with SNCA or other rare genetic variants.
Dopamine Transporter (DAT) SPECT Imaging: Positive DAT SPECT on visual inspection is necessary. Exceptions include participants with reduced DAT binding relative to age, normal range individuals with PD risk factors (including RBD, LRRK2, GBA, SNCA, or other variants), anticipating 25 % of eligible participants without a DAT deficit.

The PPMI studies are conducted in compliance with applicable regulations and ethical guidelines. Written informed consent is obtained from all participants, and the protocol has been granted approval by the local Institutional Review Boards of all participating institutions. All research activities adhere to the principles outlined in the 1964 Declaration of Helsinki and its subsequent revisions.

### 2.2 Statistical methods

We assessed the distribution of variables using histograms, Q-Q plots, and the Kolmogorov-Smirnov test. Normally distributed continuous variables were presented as mean ± SD, skewed as median (IQR), and categorical data as frequencies and percentages. Comparisons between serum potassium groups were made using chi-square/Fisher’s exact tests for categorical variables, one-way ANOVA for normal variables, and Kruskal-Wallis H test for skewed variables.

To explore the association between serum potassium levels and PPD, we performed multivariate logistic regression analyses using multiple imputation data. Serum potassium was categorized into quartiles. Covariates were selected based on clinical relevance, literature, and significant univariate results.

To assess serum potassium's continuous nature and non-linear associations, we used quartile categorization and a restricted cubic spline (RCS) model with four knots at the 5th, 35th, 65th, and 95th percentiles. We assessed non-linearity using a log-likelihood ratio test comparing a one-line model to a segmented regression model. The threshold value was determined via a two-step recursive method: First, testing 19 models with inflection points at 5 %–95 % percentiles of serum potassium levels (incremented by 5 %) to identify the highest likelihood point, defining a ±4 % range (Kmin-Kmax); Then, recursively narrowing the range by testing models at 25th, 50th, and 75th percentiles within Kmin-Kmax until the precise threshold was identified.

Subgroup analyses were conducted as prespecified. We conducted sensitivity analyses by excluding participants with any missing data, by including only those with creatinine levels <110 µmol/L, by excluding participants with diabetes and insulin use, and by excluding participants with beta-agonists use. Given that diabetes or insulin use and β-agonist use may influence serum potassium, we excluded participants with diabetes, those using insulin, and using β-agonists in sensitivity analyses to ensure robustness and avoid overfitting due to limited sample size and substantial missing data regarding diabetes status.

Analyses were done using R Statistical Software (Version 4.2.2) and Free Statistics platform (Version 1.9), with a two-sided p-value < 0.05 indicating significance.

## 3 Result

### 3.1 Population characterization

We included 1035 patients aged 64.5 ± 8.6 years, white was 94.3 %, and male were 46.7 %. Of these, the overall prevalence of PPD was 83.4 %. The baseline characteristics of the groups stratified by serum potassium are shown in Supplementary Table 1. The four groups differed in age, sex, creatinine, serum sodium, albumin (all P value < 0.05). Otherwise, the distribution of patients' characteristics (education years, race, BMI, serum glucose, ALT, AST, serum uric acid, WBC, RBC, lymphocytes, neutrophils, platelets, calcium) between serum potassium groups was similar (all P value > 0.05).

### 3.2 Multivariate analysis of serum potassium and related factors of PPD

Table 2 delineates the findings from the multivariate logistic regression analysis. Elevated serum potassium levels were associated with a heightened risk of PPD, with an odds ratio (OR) of 1.82 per unit increase (95 % confidence interval [CI], 1.04–3.19; P = 0.036), after accounting for confounding variables (Table 2).When compared to the reference group with the lowest serum potassium levels (Q1: 3.3–4.2 mmol/L), the adjusted ORs for PPD across increasing quartiles of serum potassium were as follows: Q2 (4.2–4.4 mmol/L) yielded an OR of 1.76 (95 % CI, 1.08–2.86; P = 0.022), Q3 (4.4–4.6 mmol/L) an OR of 1.91 (95 % CI, 1.14–3.2; P = 0.014), and Q4 (4.6–5.7 mmol/L) an OR of 1.88 (95 % CI, 1.17–3.02; P = 0.009), respectively.

**Table 2:** Results of multivariate logistic regression analyses of associations between serum potassium and PPD.

Variable	n. total	Model 1		Model 2		Model 3	
		OR (95 %CI)	P value	OR (95 %CI)	P value	OR (95 %CI)	P value
Serum Potassium	1035	1.93 (1.14 ∼ 3.25)	0.014	1.87 (1.07 ∼ 3.26)	0.027	1.82 (1.04 ∼ 3.19)	0.036
Quartiles							
Q1	223	1(Ref)		1(Ref)		1(Ref)	
Q2	262	1.82 (1.15 ∼ 2.86)	0.01	1.82 (1.13 ∼ 2.94)	0.014	1.76 (1.08 ∼ 2.86)	0.022
Q3	234	2.07 (1.28 ∼ 3.34)	0.003	1.97 (1.18 ∼ 3.29)	0.009	1.91 (1.14 ∼ 3.2)	0.014
Q4	316	1.97 (1.27 ∼ 3.06)	0.002	1.93 (1.21 ∼ 3.08)	0.006	1.88 (1.17 ∼ 3.02)	0.009
p for trend	1035		0.003		0.009		0.013

Model 1: unadjusted.

Model 2: adjusted age, sex, race, education years, BMI.

Model 3: adjusted Model 2, neutrophils, creatinine, serum glucose, AST, lymphocytes, total protein.

PPD, prodromal Parkinson's disease; n, number; AST, aspartate aminotransferase; BMI, body mass index.

**Fig. 2:**
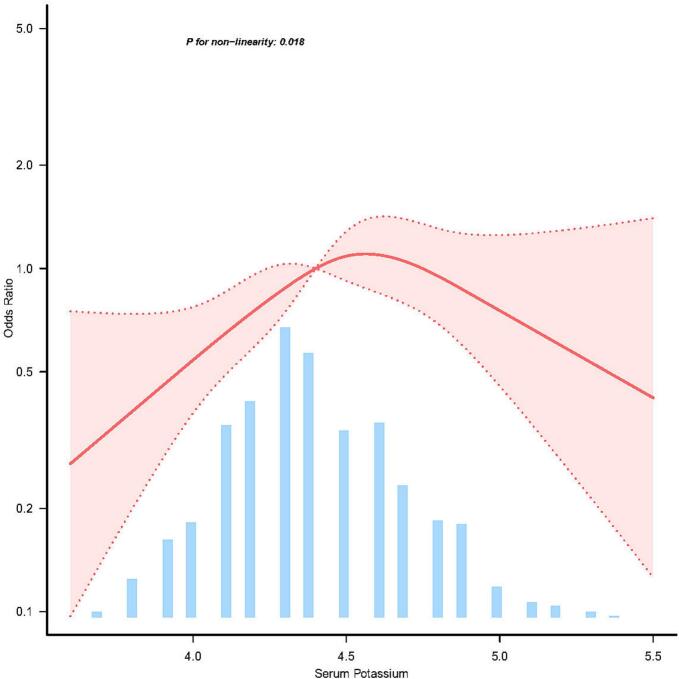
Linear dose response association between serum potassium and prodromal Parkinson's disease. Adjustment factors included age, sex, education years, race, body mass index, calcium, alanine aminotransferase, aspartate aminotransferase, lymphocytes, neutrophils, serum uric acid, serum sodium, creatinine, and serum glucose. The red line and red area represent the estimated values and their corresponding 95% confidence intervals, respectively.

The association between serum potassium and the risk of PPD, as depicted in the RCS model, revealed an inverted U-shaped curve, indicating a nonlinear association (P = 0.018) (Fig. 2). In threshold analysis, participants with serum potassium levels below 4.479 mmol/L exhibited an OR of 5.078 for PPD (95 % CI: 1.617–15.949; P = 0.0054) (Supplementary Table 3). Conversely, our analysis revealed a non-significant trend towards a decreased risk of PPD with serum potassium levels ≥4.479 mmol/L (OR = 0.384, 95 % CI: 0.103–1.431, P = 0.1538). This suggests that higher serum potassium levels may be associated with a lower risk of PPD, although this association did not reach statistical significance.

### 3.3 Subgroup analysis and sensitivity analysis

Subgroup analyses demonstrated that the association between serum potassium and PPD was robust (Supplementary Fig. 3). No significant interactions were observed among age (<65 years; ≥65 years) and sex (male and female) groups for PPD (all P for interaction >0.05). Stratified analyses by BMI (<25 kg/m^2^; 25–30 kg/m^2^; ≥30 kg/m^2^) revealed fluctuating associations between serum potassium and PPD across BMI subgroups (Supplementary Table 4), with significant differences in BMI strata for incident PPD (P = 0.023 for interaction).

In sensitivity analyses, the significant association between serum potassium and PPD were consistently maintained (Supplementary Tables 5–8).

## 4 Discussion

In this retrospective cross-sectional investigation, we initially established that serum potassium levels were significantly and independently associated to 82 % heightened risk of PPD. Notably, our analysis revealed an inverted U-shaped association between serum potassium levels and the risk of PPD, with an increased risk observed when serum potassium levels were below 4.479 mmol/L (OR = 5.078, 95 % CI: 1.617–15.949, P < 0.0054). These findings carry significant implications for the management of PPD, particularly in the white population, suggesting that potassium homeostasis may play a crucial role in disease progression and should be considered in therapeutic strategies.

Previous research has yielded mixed findings regarding the association between serum potassium and PD. One study, encompassing 120 patients with PD, reported a decrease in potassium levels in scalp hair and blood [5]. In contrast, a recent cross-sectional analysis suggested that elevated serum potassium levels constitute an independent risk factor for PD [2], with smoothed curve fitting analysis revealing a linear association between serum potassium concentration and PD risk. Our study, however, did not substantiate this linear relationship, instead identifying an inverted U-shaped pattern linking serum potassium levels to the risk of PPD. It is plausible that the serum potassium levels in our cohort were higher than those in previous studies, allowing us to discern the association between elevated serum potassium and PPD. This discrepancy may provide novel insights into the role of potassium in the PPD.

The potassium ion is an essential element for life, playing a critical role in establishing the resting membrane potential of cells, modulating the release of neurotransmitters and neuronal excitability, and preserving cellular homeostasis and volume [6]. The substantia nigra-striatum system is replete with potassium channels. Souvarish Sarkar et al. [7] suggest that potassium channels are integral to amplifying neuroinflammatory responses mediated by microglia in PD. TMEM175, identified as a lysosomal potassium channel, has been shown to modulate cellular processes. [8] Increased TMEM175 activity inhibits mitophagy, disrupting mitochondrial homeostasis and leading to an increase in reactive oxygen species (ROS) production. The subsequent accumulation of ROS further upregulates TMEM175, creating a positive feedback loop that exacerbates apoptotic cell death. In a murine model, the knockout of TMEM175 has been demonstrated to mitigate motor impairments and protect dopaminergic (DA) neurons from degeneration, highlighting the significance of TMEM175-mediated apoptosis in PD pathogenesis. The mutation and dysfunction of potassium channels are closely related to PD [9]. A previous study [6] detailed that the activation or inhibition of K+ channels, such as SK channels, A-type K+ channels, and Kv7/KCNQ channels, can alter the firing patterns of surviving substantia nigra pars compacta DA neurons and the excitability of striatal projection neurons, thereby potently alleviating motor symptoms in PD rat models.

Neurodegenerative processes have been shown to alter the integrity of blood vessels and the blood–brain barrier [10]. Reduced brain potassium in AD is paralleled by increased serum potassium levels [11]. In this paper, the association between serum potassium level and PPD is clear, but the specific causal association and the pathological mechanism of how potassium participates in PPD still need to be further studied.

Our analysis revealed a significant association between serum potassium and PPD risk only in the subgroup with BMI > 30 kg/m^2^. Higher BMI is associated with distinct metabolic profiles, including altered potassium metabolism and increased insulin resistance [12], which may modulate the relationship between serum potassium and PPD risk. The limited sample size in each BMI stratum may reduce statistical power, obscuring significant associations in other subgroups. Future studies with larger sample sizes are needed to explore this association further.

This manuscript presents several methodological strengths. Initially, our investigation of the association between serum potassium levels and PPD employed multivariate logistic regression models, which effectively controlled for potential confounders, thereby reducing bias. Additionally, we conducted smooth curve fitting and inflection point analyses to delineate the inverted U-shaped association between serum potassium and PPD, successfully identifying the critical inflection point. Furthermore, the robustness of our findings was confirmed through stratified subgroup and sensitivity analyses, which explored the serum potassium-PPD association across diverse populations.

However, this study is not without its limitations. Firstly, the observational nature of our research limits direct comparability to the gold standard of randomized controlled trials (RCTs). Secondly, the cross-sectional design precludes a comprehensive understanding of the causal mechanisms underlying the observed associations. Thirdly, our study establishes an association rather than a causal link between serum potassium and PPD, underscoring the necessity for future prospective cohort studies to affirm our observations. Fourthly, even though regression models were constructed, and stratified analyses and sensitivity analysis were performed, residual confounding effects from unmeasured or unknown factors could not be entirely excluded. Despite these constraints, rigorously designed prospective studies and mechanism investigations are imperative to substantiate our results.

## 5 Conclusions

This study highlights the association between serum potassium levels and the risk of PPD, independent of other confounding variables. Our findings suggest an inverted U-shaped association between serum potassium and PPD, with a critical threshold identified at 4.479 mmol/L. Below this threshold, the risk of PPD appears to increase with higher serum potassium levels. However, above this threshold, the risk does not further escalate with additional increases in serum potassium levels. These observations are of significant interest and may contribute to our understanding of the pathogenesis of PD and the development of potential therapeutic strategies. Further validation and confirmation of our results in larger, prospective studies are warranted to solidify these findings. Data will be accessible upon request to facilitate additional research and scrutiny.

## CRediT authorship contribution statement

**Wen Zhou:** Writing – original draft, Software, Methodology, Funding acquisition, Data curation. **Qingqing Xia:** Visualization, Data curation. **Duan Liu:** Supervision, Software. **Jun-ying Li:** Supervision, Investigation. **Liang Gong:** Writing – review & editing, Supervision, Project administration, Methodology, Funding acquisition, Conceptualization.

## Funding

This research was supported by Health China: Bu Chang Zhi Yuan Public welfare projects for heart and brain health under Grant, No. HIGHER2023073; Chengdu Medical Research Project No. 2022161; Chengdu Science and Technology Department project, No. 2024-YF05-00958-SN. Sichuan Provincial Science and Technology Department project in China, No. 2024ZYD0136.

## Declaration of competing interest

The authors declare that they have no known competing financial interests or personal relationships that could have appeared to influence the work reported in this paper.
