# Supplementary material for: Identification of inverted U-shaped curve association between serum potassium and prodromal Parkinson’s disease
Source: Clin Park Relat Disord. 2025 Apr 9;12:100323. doi: 10.1016/j.prdoa.2025.100323 (PMC12017917; doi:10.1016/j.prdoa.2025.100323)

Supplementary Figure 1 Flowchart of the study cohort.


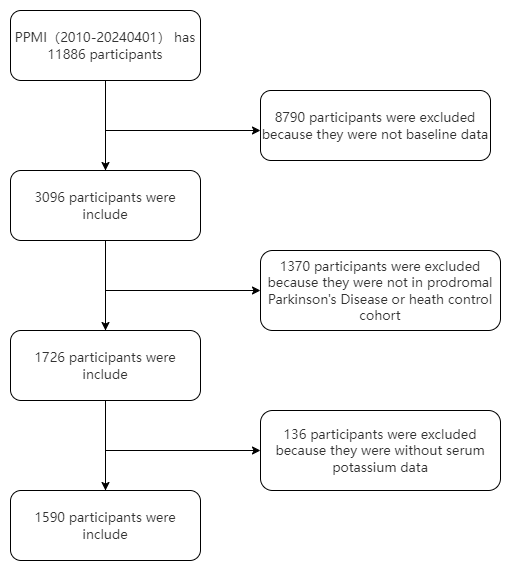


Supplementary Figure 3 Subgroup analyses of the associations between serum potassium and prodromal Parkinson's disease adjusted for age, sex, education years, race, body mass index, calcium, alanine aminotransferase, aspartate aminotransferase, lymphocytes, neutrophils, serum uric acid, serum sodium, creatinine, and serum glucose. In each case, the model was not adjusted for the stratification variable.


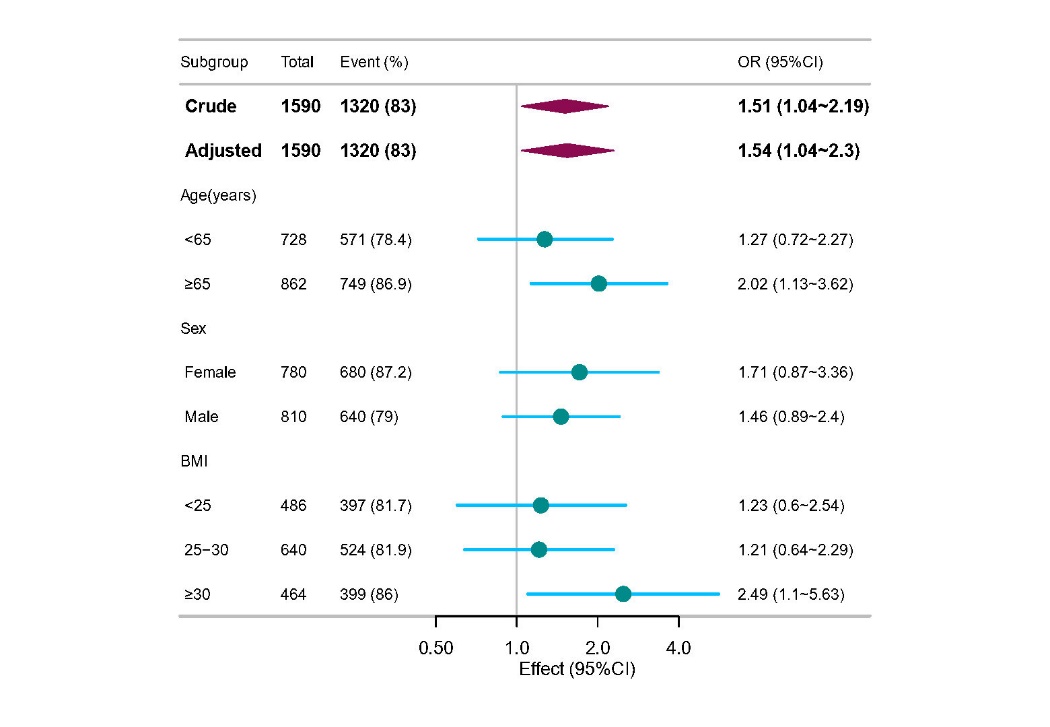

Supplement: Supplementary Data 1 [file mmc1.docx]
