# Supplementary material for: Identification of inverted U-shaped curve association between serum potassium and prodromal Parkinson’s disease
Source: Clin Park Relat Disord. 2025 Apr 9;12:100323. doi: 10.1016/j.prdoa.2025.100323 (PMC12017917; doi:10.1016/j.prdoa.2025.100323)
Supplement: Supplementary Data 2 [file mmc2.docx]

Supplementary Tabel 1 Clinical characteristics of the study population by serum potassium levels.

| Variables | Total (n = 1035) | Serum potassium | | | | p |
| --- | --- | --- | --- | --- | --- | --- |
|  |  | Q1(3.3-4.2mmol/L)  (n = 223) | Q2 (4.2-4.4mmol/L)  (n = 262) | Q3 (4.4-4.6mmol/L)  (n = 234) | Q4 (4.6-5.7mmol/L)  (n = 316) |  |
| Cohort, n (%) |  |  |  |  |  | 0.004 |
| Healthy control | 172 (16.6) | 55 (24.7) | 40 (15.3) | 32 (13.7) | 45 (14.2) |  |
| Prodromal Parkinson's disease | 863 (83.4) | 168 (75.3) | 222 (84.7) | 202 (86.3) | 271 (85.8) |  |
| Age, Mean ± SD | 64.5 ± 8.6 | 63.2 ± 9.4 | 63.6 ± 8.6 | 65.6 ± 7.9 | 65.5 ± 8.3 | 0.001 |
| Sex, n (%) |  |  |  |  |  | 0.049 |
| Female | 552 (53.3) | 129 (57.8) | 152 (58) | 117 (50) | 154 (48.7) |  |
| Male | 483 (46.7) | 94 (42.2) | 110 (42) | 117 (50) | 162 (51.3) |  |
| Education years, Mean ± SD | 16.7 ± 3.3 | 16.6 ± 3.2 | 16.8 ± 3.3 | 16.6 ± 3.4 | 16.7 ± 3.3 | 0.797 |
| Race, n (%) |  |  |  |  |  | 0.895 |
| White | 967 (94.3) | 213 (95.5) | 245 (95) | 216 (93.5) | 293 (93.6) |  |
| Black | 17 (1.7) | 4 (1.8) | 3 (1.2) | 5 (2.2) | 5 (1.6) |  |
| Asian | 5 (0.5) | 0 (0) | 2 (0.8) | 2 (0.9) | 1 (0.3) |  |
| Other (includes multi-racial) | 36 (3.5) | 6 (2.7) | 8 (3.1) | 8 (3.5) | 14 (4.5) |  |
| BMI (kg/m2), Mean ± SD | 27.4 ± 6.6 | 26.8 ± 5.1 | 27.3 ± 5.1 | 28.4 ± 9.6 | 27.1 ± 5.7 | 0.045 |
| Serum Glucose(mmol/L), Mean ± SD | 5.5 ± 1.0 | 5.6 ± 1.2 | 5.5 ± 1.0 | 5.4 ± 0.9 | 5.4 ± 0.7 | 0.054 |
| ALT(U/L), Mean ± SD | 19.0 (15.0, 25.0) | 20.0 (15.2, 26.0) | 19.0 (15.0, 25.0) | 19.0 (14.0, 25.0) | 19.0 (15.0, 25.0) | 0.535 |
| AST(U/L), Mean ± SD | 23.3 ± 12.5 | 23.8 ± 9.6 | 22.9 ± 15.3 | 22.6 ± 6.9 | 23.8 ± 14.8 | 0.641 |
| Creatinine(umol/L), Mean ± SD | 79.8 ± 16.6 | 78.5 ± 18.2 | 76.9 ± 15.0 | 81.4 ± 16.8 | 81.9 ± 16.3 | < 0.001 |
| Serum Uric Acid(umol/L), Mean ± SD | 306.3 ± 74.7 | 302.0 ± 76.5 | 306.1 ± 76.3 | 307.0 ± 70.1 | 309.0 ± 75.6 | 0.756 |
| WBC($\times$10^3^/uL), Mean ± SD | 5.9 ± 1.8 | 6.0 ± 1.9 | 6.0 ± 1.6 | 5.9 ± 1.8 | 5.8 ± 1.7 | 0.513 |
| RBC($\times$10^6^/uL), Mean ± SD | 4.6 ± 0.4 | 4.5 ± 0.4 | 4.6 ± 0.4 | 4.6 ± 0.4 | 4.6 ± 0.4 | 0.133 |
| Lymphocytes($\times$10^3^/ul), Mean ± SD | 1.7 ± 0.7 | 1.7 ± 0.6 | 1.7 ± 0.8 | 1.7 ± 0.8 | 1.6 ± 0.6 | 0.727 |
| Neutrophils($\times$10^3^/ul), Mean ± SD | 3.7 ± 1.4 | 3.8 ± 1.6 | 3.7 ± 1.2 | 3.6 ± 1.3 | 3.6 ± 1.4 | 0.494 |
| Platelets($\times$10^3^/ul), Mean ± SD | 243.5 ± 61.4 | 236.4 ± 55.7 | 245.1 ± 62.5 | 244.6 ± 60.5 | 246.4 ± 64.7 | 0.286 |
| Serum Sodium (mmol/L), Mean ± SD | 140.1 ± 2.6 | 139.8 ± 3.0 | 139.7 ± 2.4 | 140.1 ± 2.7 | 140.5 ± 2.4 | < 0.001 |
| Calcium(mmol/L), Mean ± SD | 2.4 ± 0.1 | 2.4 ± 0.1 | 2.4 ± 0.1 | 2.4 ± 0.1 | 2.4 ± 0.1 | 0.057 |
| Albumin(g/L), Mean ± SD | 44.0 ± 3.2 | 43.3 ± 3.4 | 43.9 ± 3.1 | 44.1 ± 3.1 | 44.6 ± 3.0 | < 0.001 |
| Serum Potassium(mmol/L), Mean ± SD | 4.4 ± 0.3 | 4.0 ± 0.1 | 4.3 ± 0.0 | 4.4 ± 0.0 | 4.8 ± 0.2 | < 0.001 |

N, number; HC, health control; PPD, prodromal Parkinson's disease; BMI, body mass index; RBC, red blood cell; WBC, white blood cell; ALT, alanine aminotransferase; AST, aspartate aminotransferase; race other, includes multi-racial; Numbers that do not add up to 100% are attributable to missing data.

Supplementary Table 3 Threshold effect analysis of serum potassium on PPD.

| Threshold of serum potassium | OR | 95%CI | P value |
| --- | --- | --- | --- |
| <4.479 mmol/L | 5.078 | 1.617~15.949 | 0.0054 |
| ≥4.479 mmol/L | 0.384 | 0.103~1.431 | 0.1538 |

Adjustment factors include age, sex, race, education years, BMI, neutrophils, creatinine, serum glucose, AST, lymphocytes, total protein.

AST, aspartate aminotransferase; BMI, body mass index.

Supplementary Table 4 Interactive effect of serum potassium and PPD across different BMI groups.

| variable | BMI < 25 kg/m^2^(not obese) | | BMI 25-30 kg/m^2^(overweight) | | BMI ≥ 30 kg/m^2^ (obese) | | P for interaction |
| --- | --- | --- | --- | --- | --- | --- | --- |
|  | OR 95% CI | P value | OR 95% CI | P value | OR 95% CI | P value |  |
| Serum potassium | 2.1 (0.78~5.67) | 0.141 | 1.14 (0.48~2.7) | 0.775 | 9.11 (2.21~37.63) | 0.002 | 0.023 |

Adjustment factors include age, sex, race, education years, neutrophils, creatinine, serum glucose, AST, lymphocytes, total protein.

AST, aspartate aminotransferase; BMI, body mass index.

Supplementary Table 5 Results of multivariate logistic regression analyses of associations between serum potassium and PPD in participants without missing data.

| Variable | n. total | Model 1 | | Model 2 | | Model 3 | |
| --- | --- | --- | --- | --- | --- | --- | --- |
|  |  | OR (95%CI) | P value | OR (95%CI) | P value | OR (95%CI) | P value |
| Serum Potassium | 936 | 2.29 (1.33~3.96) | 0.003 | 2.21 (1.23~3.96) | 0.008 | 2.16 (1.2~3.89) | 0.01 |
| Quartiles | | | | | | | |
| Q1 | 204 | 1(Ref) |  | 1(Ref) |  | 1(Ref) |  |
| Q2 | 234 | 1.75 (1.1~2.79) | 0.018 | 1.76 (1.08~2.88) | 0.025 | 1.69 (1.02~2.78) | 0.04 |
| Q3 | 217 | 2.19 (1.33~3.59) | 0.002 | 2.07 (1.22~3.5) | 0.007 | 2.02 (1.18~3.45) | 0.01 |
| Q4 | 281 | 2.05 (1.3~3.24) | 0.002 | 2 (1.23~3.24) | 0.005 | 1.95 (1.19~3.2) | 0.008 |
| p for trend | 936 |  | 0.002 |  | 0.006 |  | 0.008 |

Model 1: unadjusted.

Model 2: adjusted age, sex, race, education years, BMI.

Model 3: adjusted Model 2, neutrophils, creatinine, serum glucose, AST, lymphocytes, total protein.

PPD, prodromal Parkinson's disease; n, number; AST, aspartate aminotransferase; BMI, body mass index.

Supplementary Table 6 Results of multivariate logistic regression analyses of associations between serum potassium and PPD with creatine < 110 umol/L.

| Variable | n. total | Model 1 | | Model 2 | | Model 3 | |
| --- | --- | --- | --- | --- | --- | --- | --- |
|  |  | OR (95%CI) | P value | OR (95%CI) | P value | OR (95%CI) | P value |
| Serum Potassium | 992 | 2.11 (1.22~3.64) | 0.007 | 2.02 (1.13~3.63) | 0.018 | 2.14 (1.16~3.94) | 0.014 |
| Quartiles | | | | | | | |
| Q1 | 215 | 1(Ref) |  | 1(Ref) |  | 1(Ref) |  |
| Q2 | 256 | 1.78 (1.12~2.82) | 0.015 | 1.79 (1.1~2.94) | 0.02 | 1.71 (1.03~2.84) | 0.04 |
| Q3 | 224 | 1.99 (1.22~3.25) | 0.006 | 1.86 (1.1~3.14) | 0.02 | 1.95 (1.13~3.37) | 0.016 |
| Q4 | 297 | 2.05 (1.3~3.24) | 0.002 | 1.96 (1.2~3.2) | 0.007 | 1.94 (1.16~3.22) | 0.011 |
| p for trend | 992 |  | 0.003 |  | 0.011 |  | 0.012 |

Model 1: unadjusted.

Model 2: adjusted age, sex, race, education years, BMI.

Model 3: adjusted Model 2, neutrophils, creatinine, serum glucose, AST, lymphocytes, total protein.

PPD, prodromal Parkinson's disease; n, number; AST, aspartate aminotransferase; BMI, body mass index.

Supplementary Table 7 Results of multivariate logistic regression analyses of associations between serum potassium and PPD excluding participants with diabetes and insulin use.

| Variable | n. total | Model 1 | | Model 2 | | Model 3 | |
| --- | --- | --- | --- | --- | --- | --- | --- |
|  |  | OR (95%CI) | P value | OR (95%CI) | P value | OR (95%CI) | P value |
| Serum Potassium | 1023 | 1.91 (1.13~3.24) | 0.016 | 1.87 (1.07~3.27) | 0.028 | 1.82 (1.03~3.21) | 0.038 |
| Quartiles | | | | | | | |
| Q1 | 221 | 1(Ref) |  | 1(Ref) |  | 1(Ref) |  |
| Q2 | 258 | 1.86 (1.18~2.94) | 0.008 | 1.89 (1.16~3.06) | 0.01 | 1.84 (1.13~2.99) | 0.015 |
| Q3 | 231 | 2.06 (1.27~3.34) | 0.003 | 1.99 (1.19~3.31) | 0.009 | 1.95 (1.16~3.27) | 0.011 |
| Q4 | 313 | 1.97 (1.27~3.06) | 0.002 | 1.94 (1.22~3.09) | 0.005 | 1.9 (1.18~3.04) | 0.008 |
| p for trend | 1023 |  | 0.004 |  | 0.009 |  | 0.013 |

Model 1: unadjusted.

Model 2: adjusted age, sex, race, education years, BMI.

Model 3: adjusted Model 2, neutrophils, creatinine, serum glucose, AST, lymphocytes, total protein.

PPD, prodromal Parkinson's disease; n, number; AST, aspartate aminotransferase; BMI, body mass index.

Supplementary Table 8 Results of multivariate logistic regression analyses of associations between serum potassium and PPD excluding participants with beta-agonists use.

| Variable | n. total | Model 1 | | Model 2 | | Model 3 | |
| --- | --- | --- | --- | --- | --- | --- | --- |
|  |  | OR (95%CI) | P value | OR (95%CI) | P value | OR (95%CI) | P value |
| Serum Potassium | 985 | 1.82 (1.08~3.08) | 0.025 | 1.78 (1.02~3.1) | 0.044 | 1.78 (1.01~3.12) | 0.045 |
| Quartiles | | | | | | | |
| Q1 | 212 | 1(Ref) |  | 1(Ref) |  | 1(Ref) |  |
| Q2 | 255 | 1.75 (1.1~2.77) | 0.018 | 1.7 (1.05~2.77) | 0.032 | 1.63 (1~2.68) | 0.051 |
| Q3 | 215 | 1.86 (1.14~3.03) | 0.013 | 1.74 (1.03~2.92) | 0.037 | 1.7 (1.01~2.88) | 0.047 |
| Q4 | 303 | 1.91 (1.22~2.99) | 0.004 | 1.87 (1.16~3) | 0.01 | 1.85 (1.14~3) | 0.012 |
| p for trend | 985 |  | 0.008 |  | 0.017 |  | 0.019 |

Model 1: unadjusted.

Model 2: adjusted age, sex, race, education years, BMI.

Model 3: adjusted Model 2, neutrophils, creatinine, serum glucose, AST, lymphocytes, total protein.

PPD, prodromal Parkinson's disease; n, number; AST, aspartate aminotransferase; BMI, body mass index.
